# *N*,*N*′-Bis(4-methoxy­benzyl­idene)-4,4′-(*m*-phenyl­enedi­oxy)dianiline

**DOI:** 10.1107/S1600536809010939

**Published:** 2009-03-28

**Authors:** Said Nadeem, Muhammad Raza Shah, Donald VanDerveer

**Affiliations:** a HEJ Research Institute of Chemistry, International Center for Chemical & Biological Sciences, University of Karachi, Karachi 75270, Pakistan; b Molecular Structure Center, Chemistry Department, Clemson University, Clemson, SC 29634-0973, USA

## Abstract

Mol­ecules of the title compound, C_34_H_28_N_2_O_4_, a Schiff base precursor for macrocycles, are located on a mirror plane. The C=N double bond is *trans* configured. Inter­molecular C—H⋯O inter­actions stabilize the crystal packing.

## Related literature

For the importance of Schiff base macrocycles in macrocyclic and supra­molecular chemistry, see: Ali *et al.* (2008 ▶).
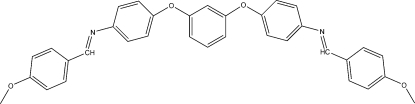


## Experimental

### 

#### Crystal data

C_34_H_28_N_2_O_4_
*M* *_r_* = 528.58
Orthorhombic, 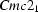

*a* = 59.344 (13) Å
*b* = 7.484 (3) Å
*c* = 5.988 (2) Å
*V* = 2659.4 (15) Å^3^
*Z* = 4
Mo *K*α radiation
μ = 0.09 mm^−1^
*T* = 163 K
0.60 × 0.41 × 0.02 mm

#### Data collection

Rigaku AFC8S Mercury CCD diffractometer
Absorption correction: multi-scan (Jacobson, 1998 ▶) *T*_min_ = 0.837, *T*_max_ = 1.000 (expected range = 0.836–0.998)
7742 measured reflections
1351 independent reflections
1164 reflections with *I* > 2σ(*I*)
*R*_int_ = 0.046

#### Refinement

*R*[*F* ^2^ > 2σ(*F* ^2^)] = 0.052
*wR*(*F* ^2^) = 0.142
*S* = 1.09
1351 reflections
185 parameters
1 restraint
H-atom parameters constrained
Δρ_max_ = 0.29 e Å^−3^
Δρ_min_ = −0.23 e Å^−3^

### 

Data collection: *CrystalClear* (Rigaku, 2001 ▶); cell refinement: *CrystalClear*; data reduction: *CrystalClear*; program(s) used to solve structure: *SHELXTL* (Sheldrick, 2008 ▶); program(s) used to refine structure: *SHELXTL*; molecular graphics: *SHELXTL*; software used to prepare material for publication: *SHELXTL*.

## Supplementary Material

Crystal structure: contains datablocks I, global. DOI: 10.1107/S1600536809010939/bt2885sup1.cif

Structure factors: contains datablocks I. DOI: 10.1107/S1600536809010939/bt2885Isup2.hkl

Additional supplementary materials:  crystallographic information; 3D view; checkCIF report

## Figures and Tables

**Table 1:** Hydrogen-bond geometry (Å, °)

*D*—H⋯*A*	*D*—H	H⋯*A*	*D*⋯*A*	*D*—H⋯*A*
C8—H8*B*⋯O7^i^	0.96	2.57	3.411 (5)	147
C13—H13⋯O17^ii^	0.96	2.52	3.405 (4)	154

Symmetry codes: (i) 
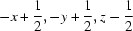
; (ii) 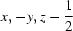.

**Fig. 1.**
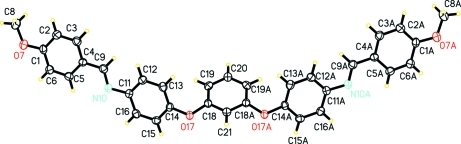
Crystal Structure of the title compound; anisotropic displacement ellipsoid plot, 50% probability; symmetry operator for generating equivalent atoms -x, y, z).

## supplementary crystallographic information

### Comment

Schiff base macrocycles have been of great importance in macrocyclic and supramolecular chemistry (Ali *et al.*,  2008). Here we report the synthesis and crystal structure of the title compound which is a precursor for the synthesis of a Schiff-base macrocycle. The crystal packing is stabilized by some short C—H···O contacts and van der Waals interactions. The methoxy group is coplanar with the benzene ring as revealed by the C6—C1—O7—C8 torsion of 175.5 (3)°. The methoxy oxygen atom   acts as an acceptor for a weak C—H···O hydrogen bond from a  neighboring molecule. C—H···O interactions stabilize the crystal packing.

### Experimental

100 mg (0.34 mmol) of 4,4'-(1,3-phenylenebis(oxy))dianiline was dissolved in 2 ml of dichloromethane and then a solution of 4-methoxybenzaldehyde (0.1 ml, 085 mmol) in 2 ml of dichloromethane was added dropwise with stirring. The reaction was stirred at 330 K for 30 min and cooled to room temperature. The solvent was then removed using a rotary evaporator to give a crude solid. The solid was dissolved in dichloromethane and slow evaporation of the dichloromethane afforded needle like crystals in 80% yield.

### Refinement

In the absence of anomalous scatterers Friedel pairs had been merged. All H atoms were geometrically fixed and allowed to ride on the corresponding non-H atom with C—H = 0.96 Å, *U*_iso_(H) = 1.5*U*_eq_(C) of the attached C atom for methyl H atoms and 1.2*U*_eq_(C) for other H atoms.

### Crystal data

**Table:**

C_34_H_28_N_2_O_4_	*D*_x_ = 1.320 Mg m^−^^3^
*M**_r_* = 528.58	Mo *K*α radiation, λ = 0.71073 Å
Orthorhombic, *C**m**c*2_1_	Cell parameters from 3132 reflections
*a* = 59.344 (13) Å	θ = 2.7–26.4°
*b* = 7.484 (3) Å	µ = 0.09 mm^−^^1^
*c* = 5.988 (2) Å	*T* = 163 K
*V* = 2659.4 (15) Å^3^	Plate, colorless
*Z* = 4	0.60 × 0.41 × 0.02 mm
*F*(000) = 1112	

### Data collection

**Table:**

Rigaku AFC8S Mercury CCD diffractometer	1351 independent reflections
Radiation source: Sealed Tube	1164 reflections with *I* > 2σ(*I*)
Graphite Monochromator	*R*_int_ = 0.046
Detector resolution: 14.6306 pixels mm^-1^	θ_max_ = 25.4°, θ_min_ = 2.7°
ω scans	*h* = −71→53
Absorption correction: multi-scan (Jacobson, 1998)	*k* = −8→9
*T*_min_ = 0.837, *T*_max_ = 1.000	*l* = −7→7
7742 measured reflections	

### Refinement

**Table:**

Refinement on *F*^2^	Primary atom site location: structure-invariant direct methods
Least-squares matrix: full	Secondary atom site location: difference Fourier map
*R*[*F*^2^ > 2σ(*F*^2^)] = 0.052	Hydrogen site location: inferred from neighbouring sites
*wR*(*F*^2^) = 0.142	H-atom parameters constrained
*S* = 1.09	*w* = 1/[σ^2^(*F*_o_^2^) + (0.0795*P*)^2^ + 2.3925*P*] where *P* = (*F*_o_^2^ + 2*F*_c_^2^)/3
1351 reflections	(Δ/σ)_max_ < 0.001
185 parameters	Δρ_max_ = 0.29 e Å^−^^3^
1 restraint	Δρ_min_ = −0.23 e Å^−^^3^

### Special details

**Table:**

Geometry. All e.s.d.'s (except the e.s.d. in the dihedral angle between two l.s. planes) are estimated using the full covariance matrix. The cell e.s.d.'s are taken into account individually in the estimation of e.s.d.'s in distances, angles and torsion angles; correlations between e.s.d.'s in cell parameters are only used when they are defined by crystal symmetry. An approximate (isotropic) treatment of cell e.s.d.'s is used for estimating e.s.d.'s involving l.s. planes.
Refinement. Refinement of *F*^2^ against ALL reflections. The weighted *R*-factor *wR* and goodness of fit *S* are based on *F*^2^, conventional *R*-factors *R* are based on *F*, with *F* set to zero for negative *F*^2^. The threshold expression of *F*^2^ > σ(*F*^2^) is used only for calculating *R*-factors(gt) *etc*. and is not relevant to the choice of reflections for refinement. *R*-factors based on *F*^2^ are statistically about twice as large as those based on *F*, and *R*- factors based on ALL data will be even larger.

### Fractional atomic coordinates and isotropic or equivalent isotropic displacement parameters (Å^2^)

**Table:**

	*x*	*y*	*z*	*U*_iso_*/*U*_eq_	
C1	0.20032 (5)	0.2529 (4)	0.2108 (8)	0.0298 (9)	
C2	0.18360 (6)	0.1751 (5)	0.0811 (7)	0.0336 (9)	
H2	0.1873	0.1211	−0.0596	0.040*	
C3	0.16140 (6)	0.1762 (5)	0.1569 (7)	0.0322 (9)	
H3	0.1497	0.1270	0.0649	0.039*	
C4	0.15600 (6)	0.2481 (4)	0.3652 (7)	0.0266 (8)	
C5	0.17305 (6)	0.3287 (4)	0.4920 (7)	0.0309 (8)	
H5	0.1695	0.3815	0.6339	0.037*	
C6	0.19483 (6)	0.3321 (5)	0.4130 (7)	0.0319 (9)	
H6	0.2064	0.3901	0.4990	0.038*	
O7	0.22265 (4)	0.2623 (4)	0.1501 (5)	0.0417 (7)	
C8	0.22934 (6)	0.1709 (7)	−0.0480 (9)	0.0566 (13)	
H8A	0.2260	0.0460	−0.0333	0.085*	
H8B	0.2452	0.1864	−0.0706	0.085*	
H8C	0.2213	0.2189	−0.1734	0.085*	
C9	0.13286 (6)	0.2372 (4)	0.4458 (7)	0.0294 (9)	
H9	0.1212	0.2054	0.3420	0.035*	
N10	0.12749 (5)	0.2679 (4)	0.6473 (6)	0.0291 (7)	
C11	0.10465 (5)	0.2522 (4)	0.7171 (7)	0.0262 (8)	
C12	0.08845 (6)	0.1478 (4)	0.6058 (7)	0.0299 (8)	
H12	0.0926	0.0816	0.4748	0.036*	
C13	0.06651 (6)	0.1403 (4)	0.6851 (7)	0.0298 (8)	
H13	0.0555	0.0692	0.6089	0.036*	
C14	0.06059 (5)	0.2357 (4)	0.8746 (6)	0.0247 (8)	
C15	0.07629 (5)	0.3366 (4)	0.9897 (7)	0.0277 (8)	
H15	0.0720	0.4020	1.1210	0.033*	
C16	0.09834 (5)	0.3414 (4)	0.9119 (7)	0.0275 (8)	
H16	0.1095	0.4075	0.9939	0.033*	
O17	0.03923 (4)	0.2212 (3)	0.9705 (4)	0.0297 (6)	
C18	0.02009 (5)	0.2435 (4)	0.8384 (7)	0.0265 (8)	
C19	0.02040 (6)	0.3185 (4)	0.6262 (7)	0.0282 (8)	
H19	0.0344	0.3466	0.5542	0.034*	
C20	0.0000	0.3517 (6)	0.5207 (9)	0.0282 (11)	
H20	0.0000	0.3988	0.3715	0.034*	
C21	0.0000	0.2007 (6)	0.9448 (10)	0.0267 (11)	
H21	0.0000	0.1432	1.0881	0.032*	

### Atomic displacement parameters (Å^2^)

**Table:**

	*U*^11^	*U*^22^	*U*^33^	*U*^12^	*U*^13^	*U*^23^
C1	0.0204 (16)	0.037 (2)	0.032 (2)	−0.0001 (13)	0.0009 (16)	0.0060 (16)
C2	0.0318 (18)	0.039 (2)	0.030 (2)	−0.0004 (15)	0.0030 (16)	−0.0016 (16)
C3	0.0286 (18)	0.0354 (19)	0.033 (2)	−0.0043 (13)	−0.0012 (17)	0.0008 (17)
C4	0.0277 (17)	0.0271 (17)	0.025 (2)	0.0007 (12)	0.0006 (15)	0.0032 (14)
C5	0.0312 (17)	0.0318 (18)	0.030 (2)	−0.0006 (13)	−0.0033 (16)	−0.0025 (16)
C6	0.0264 (16)	0.0343 (18)	0.035 (2)	−0.0021 (13)	−0.0005 (17)	−0.0011 (17)
O7	0.0250 (13)	0.0602 (17)	0.0398 (17)	−0.0013 (11)	0.0049 (13)	−0.0029 (14)
C8	0.031 (2)	0.092 (4)	0.046 (3)	0.004 (2)	0.010 (2)	−0.009 (3)
C9	0.0294 (17)	0.0305 (18)	0.028 (2)	−0.0009 (13)	−0.0015 (16)	0.0030 (17)
N10	0.0229 (13)	0.0323 (15)	0.0321 (19)	−0.0009 (11)	0.0007 (14)	−0.0020 (14)
C11	0.0230 (16)	0.0260 (17)	0.030 (2)	0.0013 (12)	0.0016 (16)	0.0015 (15)
C12	0.0281 (17)	0.0298 (17)	0.032 (2)	0.0029 (13)	0.0002 (16)	−0.0040 (17)
C13	0.0255 (16)	0.0296 (16)	0.034 (2)	−0.0031 (13)	−0.0010 (16)	−0.0008 (16)
C14	0.0205 (15)	0.0254 (16)	0.028 (2)	0.0018 (12)	0.0004 (15)	0.0057 (14)
C15	0.0318 (17)	0.0255 (16)	0.0257 (19)	0.0025 (12)	−0.0012 (15)	−0.0004 (15)
C16	0.0265 (15)	0.0254 (16)	0.031 (2)	−0.0001 (12)	−0.0031 (15)	−0.0005 (15)
O17	0.0207 (11)	0.0401 (13)	0.0282 (16)	0.0032 (9)	−0.0002 (11)	0.0020 (12)
C18	0.0223 (17)	0.0264 (15)	0.031 (2)	0.0007 (12)	−0.0016 (14)	−0.0034 (16)
C19	0.0284 (17)	0.0260 (15)	0.030 (2)	−0.0004 (12)	0.0051 (15)	0.0006 (16)
C20	0.028 (2)	0.026 (2)	0.030 (3)	0.000	0.000	0.001 (2)
C21	0.028 (2)	0.025 (2)	0.027 (3)	0.000	0.000	0.002 (2)

### Geometric parameters (Å, °)

**Table:**

C1—O7	1.376 (4)	C11—C12	1.407 (5)
C1—C6	1.387 (6)	C12—C13	1.387 (4)
C1—C2	1.388 (5)	C12—H12	0.9600
C2—C3	1.394 (5)	C13—C14	1.386 (5)
C2—H2	0.9600	C13—H13	0.9600
C3—C4	1.395 (6)	C14—C15	1.384 (5)
C3—H3	0.9600	C14—O17	1.396 (4)
C4—C5	1.402 (5)	C15—C16	1.390 (5)
C4—C9	1.458 (5)	C15—H15	0.9600
C5—C6	1.377 (5)	C16—H16	0.9600
C5—H5	0.9600	O17—C18	1.394 (4)
C6—H6	0.9600	C18—C19	1.389 (6)
O7—C8	1.425 (6)	C18—C21	1.389 (4)
C8—H8A	0.9599	C19—C20	1.388 (4)
C8—H8B	0.9599	C19—H19	0.9600
C8—H8C	0.9599	C20—C19^i^	1.388 (4)
C9—N10	1.269 (5)	C20—H20	0.9600
C9—H9	0.9600	C21—C18^i^	1.389 (4)
N10—C11	1.423 (4)	C21—H21	0.9600
C11—C16	1.395 (5)		
			
O7—C1—C6	115.8 (3)	C16—C11—N10	117.5 (3)
O7—C1—C2	124.2 (4)	C12—C11—N10	123.9 (3)
C6—C1—C2	120.0 (3)	C13—C12—C11	120.1 (3)
C1—C2—C3	119.4 (4)	C13—C12—H12	119.9
C1—C2—H2	120.3	C11—C12—H12	119.9
C3—C2—H2	120.3	C14—C13—C12	119.8 (3)
C2—C3—C4	120.7 (3)	C14—C13—H13	120.1
C2—C3—H3	119.6	C12—C13—H13	120.1
C4—C3—H3	119.6	C15—C14—C13	121.2 (3)
C3—C4—C5	119.0 (3)	C15—C14—O17	116.7 (3)
C3—C4—C9	119.4 (3)	C13—C14—O17	121.8 (3)
C5—C4—C9	121.7 (3)	C14—C15—C16	118.8 (3)
C6—C5—C4	120.0 (4)	C14—C15—H15	120.6
C6—C5—H5	120.0	C16—C15—H15	120.6
C4—C5—H5	120.0	C15—C16—C11	121.4 (3)
C5—C6—C1	120.8 (3)	C15—C16—H16	119.3
C5—C6—H6	119.6	C11—C16—H16	119.3
C1—C6—H6	119.6	C18—O17—C14	119.8 (3)
C1—O7—C8	117.6 (3)	C19—C18—C21	121.6 (3)
O7—C8—H8A	109.5	C19—C18—O17	123.8 (3)
O7—C8—H8B	109.5	C21—C18—O17	114.3 (4)
H8A—C8—H8B	109.5	C20—C19—C18	118.5 (3)
O7—C8—H8C	109.5	C20—C19—H19	120.7
H8A—C8—H8C	109.5	C18—C19—H19	120.7
H8B—C8—H8C	109.5	C19—C20—C19^i^	121.4 (5)
N10—C9—C4	122.8 (3)	C19—C20—H20	119.3
N10—C9—H9	118.6	C19^i^—C20—H20	119.3
C4—C9—H9	118.6	C18^i^—C21—C18	118.3 (5)
C9—N10—C11	120.3 (3)	C18^i^—C21—H21	120.9
C16—C11—C12	118.6 (3)	C18—C21—H21	120.9
			
O7—C1—C2—C3	−178.7 (3)	N10—C11—C12—C13	−179.8 (3)
C6—C1—C2—C3	−0.6 (5)	C11—C12—C13—C14	−0.2 (5)
C1—C2—C3—C4	−2.6 (5)	C12—C13—C14—C15	−1.1 (5)
C2—C3—C4—C5	3.6 (5)	C12—C13—C14—O17	−174.9 (3)
C2—C3—C4—C9	−176.2 (3)	C13—C14—C15—C16	0.1 (5)
C3—C4—C5—C6	−1.6 (5)	O17—C14—C15—C16	174.1 (3)
C9—C4—C5—C6	178.2 (3)	C14—C15—C16—C11	2.3 (5)
C4—C5—C6—C1	−1.5 (5)	C12—C11—C16—C15	−3.5 (5)
O7—C1—C6—C5	−179.1 (3)	N10—C11—C16—C15	178.6 (3)
C2—C1—C6—C5	2.6 (5)	C15—C14—O17—C18	134.4 (3)
C6—C1—O7—C8	175.3 (4)	C13—C14—O17—C18	−51.6 (4)
C2—C1—O7—C8	−6.4 (5)	C14—O17—C18—C19	−15.0 (4)
C3—C4—C9—N10	166.3 (3)	C14—O17—C18—C21	171.2 (3)
C5—C4—C9—N10	−13.6 (5)	C21—C18—C19—C20	0.4 (5)
C4—C9—N10—C11	−179.0 (3)	O17—C18—C19—C20	−173.0 (3)
C9—N10—C11—C16	−159.2 (3)	C18—C19—C20—C19^i^	2.6 (6)
C9—N10—C11—C12	23.0 (5)	C19—C18—C21—C18^i^	−3.3 (6)
C16—C11—C12—C13	2.4 (5)	O17—C18—C21—C18^i^	170.7 (3)

Symmetry codes: (i) −*x*, *y*, *z*.

### Hydrogen-bond geometry (Å, °)

**Table:**

*D*—H···*A*	*D*—H	H···*A*	*D*···*A*	*D*—H···*A*
C8—H8B···O7^ii^	0.96	2.57	3.411 (5)	147
C13—H13···O17^iii^	0.96	2.52	3.405 (4)	154

Symmetry codes: (ii) −*x*+1/2, −*y*+1/2, *z*−1/2; (iii) *x*, −*y*, *z*−1/2.
